# A232 RISK OF TOTAL METACHRONOUS ADVANCED NEOPLASIA AT SURVEILLANCE COLONOSCOPY AFTER DETECTION OF SERRATED LESIONS: A MATCHED COHORT STUDY

**DOI:** 10.1093/jcag/gwac036.232

**Published:** 2023-03-07

**Authors:** R Djinbachian, M -L Lafontaine, J C Anderson, H Pohl, T Dufault, M Boivin, M Bouin, D von Renteln

**Affiliations:** 1 CHUM, Montreal, Canada; 2 Dartmouth Geisel School of Medicine, Hanover; 3 CHUM, Montreal, United States

## Abstract

**Background:**

Serrated lesions (SLs), including sessile serrated lesions (SSL) and traditional serrated adenomas (TSA) have become subject of increased interest for their role as CRC precursors.

**Purpose:**

Study aim was to evaluate the risk to develop total metachronous advanced neoplasia (T-MAN) at follow-up in patients with index SL compared to a matched cohort without SL.

**Method:**

Patients 45-74y with SLs were identified through pathology database search. SL patients were matched 2:1 by sex; age; synchronous polyps (high-risk adenoma [HRA], low-risk adenoma [LRA], no-adenoma); timing of index, to patients without SL. Primary outcome was risk of T-MAN (advanced adenoma or high-risk SL) at follow-up. Secondary outcomes included risk of T-MAN stratified by synchronous polyps and SL characteristics.

**Result(s):**

1425 patients were included (475 patients, 642 SLs; 950 controls (mean follow-up 2.9 vs 3.9y). The SL group had greater risk of T-MAN compared to the non-SL group [Hazard-ratio (HR)=6.12 (95%confidence-interval (CI)3.91-9.58)]. Patients with SL+HRA had higher risk of T-MAN compared to HRA alone [HR=2.62 (95%CI 1.45-4.71)], as well as patients with SL+LRA compared to LRA alone [HR=7.03 (95%CI 2.78-18.44)], and SL without adenoma compared to no-adenoma [HR=14.87 (95%CI 6.51-33.95)]. Presence of proximal SSL (HR=9.30), large SSL (HR=17.87) and proximal large SSL (HR=24.99), but not distal SSL, was associated with greater risk for T-MAN.

**Conclusion(s):**

Patients with SLs are at greater risk for developing T-MAN regardless of synchronous adenomas. Patients with SL and HRA, and those with large or proximal SSLs appear to be at greatest risk for T-MAN.

**Please acknowledge all funding agencies by checking the applicable boxes below:**

Other

**Please indicate your source of funding;:**

ACG

**Disclosure of Interest:**

R. Djinbachian Grant / Research support from: Grant from the American College of Gastroenterology for the conduction of this project, M.-L. Lafontaine: None Declared, J. Anderson: None Declared, H. Pohl: None Declared, T. Dufault: None Declared, M. Boivin: None Declared, M. Bouin: None Declared, D. von Renteln Grant / Research support from: Daniel von Renteln is supported by a “Fonds de Recherche du Québec Santé” (FRQS) career development award and has received research funding from ERBE, Ventage, Pendopharm, Fujifilm, and Pentax., Consultant of: Boston Scientific and Pendopharm,

